# Exploring Nunavut Public Health System's Readiness to Implement Obesity Prevention Policies and Programs in the Canadian Arctic

**DOI:** 10.1155/2019/1584956

**Published:** 2019-05-09

**Authors:** Victor O. Akande, Robert A. C. Ruiter, Stef P. J. Kremers

**Affiliations:** ^1^Maastricht University, Department of Health Promotion, NUTRIM School of Nutrition and Translational Research in Metabolism, Medical Center+, P.O. Box 616, 6200 MD, Maastricht, Netherlands; ^2^Maastricht University, Department of Work & Social Psychology, Faculty of Psychology & Neuroscience, Universiteitssingel 40, Maastricht 6200 MD, Netherlands

## Abstract

**Background:**

Rapid changes in the food and built environments in the Canadian Arctic have contributed to a dramatic increase in the prevalence rates of obesity. The objective of this study was to explore the determinants of Nunavut public health system's commitment to implement obesity prevention policies and programs in the territory to reduce the burden of obesity-related diseases.

**Methods:**

In total, 93 program managers, program officers, and policy analysts who are responsible for program and policy development and implementation within the Nunavut Department of Health (NDH) were asked to complete the validated Organizational Readiness for Implementing Change (ORIC) questionnaire. Organization-level readiness (commitment) was determined based on aggregated individual-level data using bivariate correlations and multivariate linear regression analyses.

**Results:**

Of the 93 questionnaires that were distributed only 67 (72%) were returned fully completed. Organization-level commitment to implement obesity prevention policies and programs was low. Only 2.9% of respondents strongly agreed that NDH was committed to implementing obesity prevention policies and programs. The study showed a strong positive correlation between NDH's commitment and perceived value (r = .73), perceived efficacy (r = .50), and resource availability (r = .25). There was no correlation between commitment and knowledge. In the multivariate linear regression model, perceived value was the only significant predictor of NDH's commitment to implement obesity prevention policies and programs (*β* = 0.66).

**Conclusions:**

Successful adoption and implementation of obesity prevention policies and programs in the Canadian Arctic largely depend on the perception of value and benefits of and belief in the change efforts among employees of the Nunavut Department of Health. Convincing policy makers of the value of preventive policies and programs is an important and necessary first step towards decreasing the prevalence of obesity in the Inuit population.

## 1. Background

Canadian Inuit have been undergoing rapid lifestyles transition in the last five decades [1]. The shift in patterns of food consumption and physical activity has led to the development of the so-called “energy balance-related” health problems such as diabetes and cardiovascular diseases [2, 3]. The rates of overweight and obesity in the population are particularly alarming. For example, Egeland et al. [4] reported that 29% of Inuit men and 41.6% of Inuit women are obese. The prevalence of abdominal obesity, measured by waist circumference, was particularly high among Inuit women at 59.8% compared to 40%, nationally. An increasing body of evidence suggests that unhealthy behaviors such as the consumption of energy-dense food and sedentary behavior have resulted in significant weight gain and are major drivers of the rising rates of diet-sensitive chronic diseases [5–8]. The development and implementation of effective solutions to the growing problem require concerted efforts from the health systems organization in Nunavut. Moreover, findings from research conducted in healthcare settings suggest that assessing an organization's readiness to implement change efforts is of interventionistic importance [9–11].

According to the World Health Organization [12], obesity remains a formidable but preventable risk factor for a number of life-altering chronic diseases. In Nunavut, the prevalence of Diabetes Type II was about 1% in 2002 and had increased to 4.4% by 2008/2009 [13]. As a result, there is a growing concern among health professionals about the rising rates of obesity-related problems in the population. This has led to increasing calls for more focused health promotion interventions to address the trending public health problem [14–17]. The direct and indirect costs of obesity have equally become causes of concern amongst healthcare professionals, with an estimated share in excess of 6% of total healthcare costs in many countries [18]. In Canada, the economic burden of obesity is estimated to range from $4.6 billion to $7.1 billion annually [19]. Apart from the effects on physical health, psychosocial impacts of excessive weight gain on individuals and families are well documented among Inuit [20, 21] and other populations [22]. A large proportion of obese individuals have low self-esteem and face negative attitudes and stereotypes in a variety of settings such as work, school, the social media, and within the healthcare system [19].

Despite the increasing rates of obesity in Nunavut Inuit population and the attendant physical, psychosocial, and economic impacts, the extent to which Nunavut Department of Health (NDH) is committed to implementing change efforts is not known. This has not been previously examined. Evidence from past literatures suggests that relatively little is known about factors influencing implementation of change efforts within healthcare organizations [23, 24]. Change commitment is integral to successful implementation of an effective intervention. According to Weiner, both change commitment and change efficacy determine readiness. Weiner defined change efficacy as a measure of perceived collective capability of an organization for the desired change. The organizational will to fight the “wicked problem” [25] appears to remain elusive to the responsible government departments across Canada, including the Government of Nunavut Department of Health (NDH). The mandate of NDH includes the implementation of change initiatives which are developed to address the upstream risk factors of obesity as well as the health and psychosocial impacts.

According to Stokols [26], there has been an increasing shift in focus from individually oriented analysis of health behaviour to “behavioural strategies of health promotion with efforts to strengthen environmental supports within the broader community that are conducive to personal and collective wellbeing” (p. 282). Efforts required to strengthen certain elements in the environment are often beyond an individual's capacity. For example, research has shown that organizational readiness for change is a critical factor for successful and sustained implementation of change initiatives [10, 27, 28]. Government policies and regulations are upstream factors that control the food environment, physical activity, and healthcare systems in general terms. All these elements directly and indirectly impact on choices, accessibility, affordability, and, consequently, obesity rates in the population.

In the current study, we examined the commitment of NDH as an upstream macroenvironmental factor influencing the implementation of effective and appropriate obesity prevention policies and programs in the Canadian Arctic. This government department is mandated to formulate health policies and standards, including implementing initiatives such as obesity prevention programs and services. This study therefore examined the collective organizational readiness of the NDH to implement obesity prevention policies and programs in the territory. To date, research on the topic has largely focused on individual readiness and less on collective readiness at organizational level [11]. Research is needed to advance our knowledge on organizational readiness and increase our understanding on the subject. Our study sought to provide evidence-based guidance on determinants of collective readiness at NDH and, particularly, elucidate elements that might enhance the organizational readiness for implementing obesity prevention policies and programs, given the impacts of obesity on the quality of life of affected individuals in Nunavut.

To explore NDH's commitment to implementing obesity prevention policies and programs in view of the rate in the territory, we utilized a validated instrument called Organizational Readiness for Implementing Change (ORIC), developed by Shea et al. [11]. This measure was used to assess whether NDH organizational members are “psychologically and behaviourally prepared to implement the change” [11] required to reduce the growing epidemic of obesity in the territory.

### 1.1. Organizational Readiness for Implementing Change (ORIC) Measure

**Figure 1:**
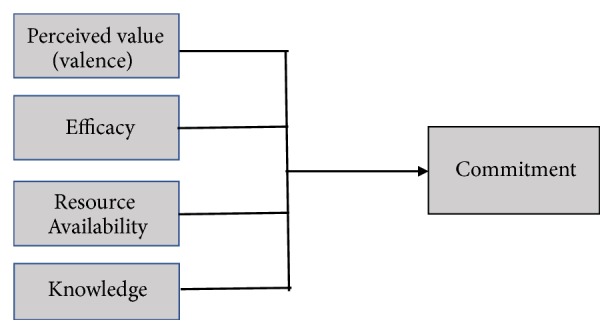
Research model explaining the relationships between NDH's readiness (change commitment) and the determining factors.

The measure of organizational readiness is a psychometric instrument that is underpinned by Weiner's theory of organization readiness for change [11, 28]. According to Weiner, organizational readiness is a multilevel and multifaceted construct that can be measured at individual level or collectively (at team, departmental, and organizational levels) in a healthcare organization that is responsible for implementing change efforts. ORIC is based on the assumption that the successful implementation of innovative solutions often requires collective and well-coordinated actions by employees of an organization [11]. In the current study, we group-referenced items, for example, using “we know….” instead of “I know….” as previously described [11] to pivot participants' attention and responses on the team's collective readiness rather than personal readiness of individuals. In the study, we administered a 30-item ORIC questionnaire using a five-point Likert scale that ranged from “strongly disagree” to “strongly agree.” Since ORIC is a multifaceted construct, we examined four facets of change commitment according to the model (Figure 1) and their determinants.

### 1.2. Nunavut and Research Context

Nunavut is located in the Arctic Region of Canada. The territory covers an extensive area of 2,093,190 km^2^, accounting for 21% of Canada's land and freshwater area [29]. Nunavut is unique compared to other jurisdictions in Canada owing to the remoteness of its 25 communities in the Arctic, the dispersion of the small population of approximately 38,243 [30], and its total reliance on air transportation. All these combine to make healthcare delivery a very challenging task [31]. Households in Nunavut rely on air transportation with its attendant prohibitive costs in order to access essential commodities such as medical, food, and other household supplies. Accessibility and availability of foods and medical supplies are therefore impacted by this mode of transportation except in the summer when nonperishable supplies are sealifted [32].

The Government of Nunavut Department of Health is responsible for delivering healthcare services in the territory, including developing policies and legislations that govern the healthcare system and programs for prevention of illnesses and elimination or reduction of risk factors of diseases including obesity.

## 2. Methods

### 2.1. Data Collection

Ethical approval was obtained from the Ethical Review Committee of Psychology and Neuroscience at Maastricht University, Netherlands (Reference# ECP-148 05_03_2015) as well as through a research license from the Nunavut Research Institute (License# 050 1315-Amended). An initial pilot was conducted with a random sample of 10 volunteers who were program officers and policy analysts in the Department of Health. The purpose of the study was discussed with the volunteers who later reviewed the research questions, completed the questionnaires, and provided comments. In response to the feedback, we provided definitions of terms used in the facets of change commitment being explored: valence or perceived value (intrinsic attractiveness of the change efforts and to what extent the change valued is); efficacy (shared belief in the employees' collective ability to engage in a course of action that will lead to change); resource availability (collective perception by employees that resources needed to implement are available, including fund, personnel, equipment, and infrastructure); and knowledge (perceived knowledge about resources, time, and tasks requirements for implementing the desired change). Further, we provided examples of healthy public policies and both conventional and innovative approaches that have either been adopted by regional and national governments or proposed by obesity prevention experts: increasing taxes on junk foods and subsidies on healthy foods [33], fruit and vegetable initiatives, active living policies, social marketing campaigns [12], and increasing access to, and availability of, traditional foods and on-the-land/water activities including hunting, trapping, and fishing to promote active living [32]. The descriptions and examples were included to increase the participants' understanding of the purpose of the study and thus increase the reliability of the data provided.

The ORIC questionnaire was distributed to all public/population health and health policy personnel who were responsible for policy or program development, implementation, and evaluation in the Department of Health. Of the 93 questionnaires that were distributed only 67 were returned and fully completed across relevant divisions within the NDH.

### 2.2. Data Analysis

Following collection, data were entered in IBM SPSS Statistics Version 24 for cleaning and subsequent analyses. Data were analyzed using descriptive statistics, Pearson's correlations, and multivariate linear regressions. Missing values were imputed by the item means. Scores for the study variables were checked for normal distributions using tests for skewness and kurtosis [34]. Further, test for regression diagnostics for outliers were conducted as recommended by Fox [35]. Descriptive analyses included the mean and standard deviation of score values. Subscale scores were computed by summing the scores on the respective subscales: the higher the score, the more agreeable the respondent to, for instance, the NDH's commitment to change efforts.

Internal consistency analyses of the constructs of organizational readiness revealed good psychometric properties according to their Cronbach alpha (*α*) values except the concept of knowledge. The alpha (*α*) values of the concepts were change commitment = .91; perceived value .85; efficacy = .83; resource availability = .61; and knowledge = -.29. Given the poor reliability of the knowledge factor, we decided to use each of the three items (knowledge-time, knowledge-resources, and knowledge-actions) that constituted the factor as an independent single item subscale. Associations with organization-level readiness (commitment) were determined based on aggregated individual-level data using bivariate correlations and multivariate linear regression analyses.

## 3. Results

67 questionnaires were fully completed for analysis, representing 72% of the public health workforce. Of these, 82% were women and 18% were men, approximately reflecting gender distribution in the Department of Health's workforce. Our findings showed that only 2.9% and 35.7% of respondents strongly agreed and agreed, respectively, with the statement that “we are committed to implementing obesity prevention policies and programs.” On the other hand, 28.6% of respondents disagreed or strongly disagreed with the statement. Another 28.6% of respondents neither agreed nor disagreed that NDH was committed to implementing obesity prevention policies and programs.

Relationships between the variables were examined (Table 1). Strong positive correlations were observed between change commitment and perceived value, efficacy, and resource availability. Additionally, there was evidence of a positive association between commitment and two knowledge variables, knowledge-time and knowledge-actions. There was a strong positive relationship between perceived value and two variables—efficacy and resource availability. There were strong positive relationships between efficacy and resource availability as well as the knowledge-actions. In addition, a strong positive correlation was observed between resource availability and knowledge-actions. However, there was no significant relationship between commitment and knowledge-resources and perceived value. No significant relationships were observed between efficacy and knowledge-time and knowledge-resources.

In the multivariate linear regression model, perceived value and the knowledge-time were strongly correlated (Table 2) with NDH's readiness for implementing obesity prevention policies and programs (standardised beta = .66; p<.01 and standardised beta = .18; p<.04, respectively).

## 4. Discussion

The study examined the degree of NDH's commitment to the implementation of effective and appropriate obesity prevention policies and programs. Results showed that organization-level commitment to implementing obesity prevention policies and programs was generally low. Research evidence in the field has suggested that a lack of organizational readiness for change may account for as many as half of all unsuccessful change initiatives in a variety of fields including healthcare. This often necessitates a redesign of intervention efforts [36–38] in the face of limited resources.

Our results indicated that only 2.9% of the employees were very confident of their organization's commitment to implementing obesity prevention policies and programs, and approximately one-third of the employees were somewhat confident. Our findings demonstrated that perceived value, efficacy, and resource availability were positively associated with NDH's commitment to implementing obesity prevention policies and programs in Nunavut. This is in line with Weiner's theory of organizational readiness for change which postulates that change commitment and perceived capacity for desired change (change efficacy) combine to determine organizational readiness [27, 28]. These are subject to how favourably organizational members perceive and evaluate the implementation capability and associated tasks (perceived value), resource availability, and other inherent factors that are likely to facilitate or hinder change efforts such as leadership commitment and environmental factors [39].

The large number of individuals surveyed relative to the numerical strength of the employees in the lines of work, the high response rate, and the gathering of independent responses from employees in the lower and management cadres within NDH can all be considered as strengths of the study. Our research provides preliminary empirical confirmation of Weiner's framework of organizational readiness. The findings also provide a strong basis for a more extensive investigation given the importance and relevance of organizational readiness to a successful and sustainable implementation of changed efforts in healthcare organizations. In the context of NDH, it is important for the organization to critically examine the underlying factors mediating the abysmally low organizational commitment to implementing obesity prevention policies and programs.

The responses obtained from the NDH employees showed low efficacy and perceived value, suggesting that the collective capability to engage in a course of action that will lead to change is suboptimal. The perceived collective value of the implementation efforts appears to be lower than what is needed to move changed efforts in a positive direction. The low perceived value on obesity prevention efforts may be linked to a more focused attention on other pressing social/health issues that were prioritized locally during the community-based needs assessment and priority setting exercises in the face of limited resources. Findings from the needs assessment (unpublished) indicated that while obesity was not identified as a priority, food insecurity, alcohol abuse, and tobacco smoking were ranked as top three priorities in many communities and are currently receiving considerable attention from NDH. The limited community-level support may have combined with other factors including financial limitations to trigger suboptimal efforts on obesity prevention.

Our results call for the redesign of intervention efforts that focus on eliminating barriers and promoting facilitators of changed efforts. For example, collective perception by employees that resources needed to implement required policies and programs as well as other situational factors must be fully analyzed and appropriate elements incorporated in the redesign of an intervention strategy.

Organizational readiness is a team rather than individual effort. Lehman et al. [40] described the construct as collective perceptions of institutional resources, the prevailing organizational culture and climate, and motivational readiness. Thus, the fundamental contextual factors of culture and climate need to be examined in more detail to understand the proximal explanatory role of perceived value and knowledge. Convincing policy makers of the perceived value of preventive policies and programs is an important and necessary first step towards decreasing the prevalence of obesity in the Inuit population. But it is likely that such efforts will increase in effectiveness when additional energy is put in understanding and changing the policy context in NDH.

## 5. Conclusions

Canadian Arctic has undergone significant social and environmental changes resulting in the disruption of Nunavut Inuit traditional ways of life. The changes have reduced the reliance on traditional food gathering and processing activities and increased dependence on energy-dense store-bought foods and motorized transportation. As a consequence, many Nunavut Inuit have become overweight or obese and developed diet-sensitive chronic diseases. Although these sociocultural and environmental changes cannot be reversed or stopped, opportunities exist to explore policy and program interventions for the population. Despite the severity of the problem and the urgent need to identify effective solutions, findings from the current study suggest that the collective capacity within the NDH to effectively respond by developing and implementing interventions is low. Efforts should therefore focus on how employees' perceived value and efficacy can be improved to translate implementation efforts into tangible health and psychosocial outcomes.

## Figures and Tables

**Table 1:** Mean scores, standard deviations, and bivariate Pearson correlations for study variables (n=67).

Variables	Mean (SD)	1	2	3	4	5	6	7
1. Commitment	15.15 (4.16)	1						

2. Perceived value	41.28 (5.39)	.73*∗∗*	1					

3. Efficacy	21.87 (4.39)	.50*∗∗*	.55*∗∗*	1				

4. Resource availability	16.26 (2.62)	.25*∗∗*	.34*∗∗*	.60*∗∗*	1			

5. Knowledge-time	2.42 (.78)	.27*∗*	.10	.22	−.02	1		

6. Knowledge-resources	4.03 (.65)	.07	−.02	.02	.02	−.14	1	

7. Knowledge-actions	3.61 (.82)	.26*∗*	.29*∗*	.41*∗∗*	.30*∗*	.07	−.21	1

*∗*P < 0.05; *∗∗*P<0.01

**Table 2:** Standardised regression coefficients, *P* values, and explained variance from regression analysis for organizational readiness (commitment) to implementing obesity prevention policies and programs (N=67).

Predictor variable	*β*	*P* value	R^2^
Perceived value	.66	<.001	.60
Efficacy	.11	.40	
Resource Availability	-.06	.58	
Knowledge-time	.18	.04	
Knowledge-resources	.11	.19	
Knowledge-actions	.06	.54	

## Data Availability

The datasets used and/or analyzed during the current study are available from the corresponding author on reasonable request.
